# Insights into the vaginal microbiome in a diverse group of women of African, Asian and European ancestries

**DOI:** 10.7717/peerj.14449

**Published:** 2022-11-29

**Authors:** Orville St. E. Roachford, Angela T. Alleyne, Karen E. Nelson

**Affiliations:** 1 Department of Biological and Chemical Sciences, The University of the West Indies, Cave Hill Campus, Bridgetown, Barbados; 2 J. Craig Venter Institute, Rockville, MD, United States of America; 3 J. Craig Venter Institute, La Jolla, CA, United States of America

**Keywords:** Vaginal microbiome, Community state types, CST subtypes, Ethnicity, Ancestries, *Lactobacillus*, Pathobionts, *Mycoplasma*, *Prevotella*, *Gardnerella*

## Abstract

**Background:**

Intra-continentally, vaginal microbiome signatures are reported to be significantly different between Black and Caucasian women, with women of African ancestry having the less well defined heterogenous bacterial community state type (CST) deficient of *Lactobacillus* species (CST IV). The objective of this study was to characterize the vaginal microbiomes across a more diverse intercontinental group of women (*N* = 151) of different ethnicities (African American, African Kenyan, Afro-Caribbean, Asian Indonesian and Caucasian German) using 16S rRNA gene sequence analysis to determine their structures and offer a comprehensive description of the non-*Lactobacillus* dominant CSTs and subtypes.

**Results:**

In this study, the bacterial composition of the vaginal microbiomes differed significantly among the ethnic groups. *Lactobacillus* spp. (*L*. *crispatus* and *L*. *iners*) dominated the vaginal microbiomes in African American women (91.8%) compared to European (German, 42.4%), Asian (Indonesian, 45.0%), African (Kenyan, 34.4%) and Afro-Caribbean (26.1%) women. Expanding on CST classification, three subtypes of CST IV (CST IV-A, IV-B and IV-C) (*N* = 56, 37.1%) and four additional CSTs were described: CST VI *Gardnerella vaginalis*—dominant (*N* = 6, 21.8%); CST VII (*Prevotella*—dominant, *N* = 1, 0.66%); CST VIII (*N* = 9, 5.96%), resembling aerobic vaginitis, was differentiated by a high proportion of taxa such as *Enterococcus*, *Streptococcus* and *Staphylococcus* (relative abundance [RA] > 50%) and CST IX (*N* = 7, 4.64%) dominated by genera other than *Lactobacillus*, *Gardnerella* or *Prevotella* (*e.g*., *Bifidobacterium breve* and *Anaerococcus vaginalis*). Within the vaginal microbiomes, 32 “taxa with high pathogenic potential” (THPP) were identified. Collectively, THPP (mean RA ~5.24%) negatively correlated (r_s_ = −0.68, *p* < 2.2e−16) with *Lactobacillus* species but not significantly with *Gardnerella*/*Prevotella* spp. combined (r = −0.13, *p* = 0.1). However, at the individual level, *Mycoplasma hominis* exhibited moderate positive correlations with *Gardnerella* (r = 0.46, *p* = 2.6e−09) and *Prevotella* spp. (r = 0.47, *p* = 1.4e−09).

**Conclusions:**

These findings while supporting the idea that vaginal microbiomes vary with ethnicity, also suggest that CSTs are more wide-ranging and not exclusive to any particular ethnic group. This study offers additional insight into the structure of the vaginal microbiome and contributes to the description and subcategorization of non-*Lactobacillus-*dominated CSTs.

## Introduction

The vaginal microbiome is thought to be influenced by host genetics, environmental and cultural factors (Gupta, Paul & Dutta, 2017). Whereas the microbial composition of the vaginal microbiome is individual-specific and similar across non-Caucasian groups including Asian, Black and Hispanic women (Feehily et al., 2020; Si et al., 2017; Zhou et al., 2010), the structure of the vaginal microbial communities of women of African American descent has been reported to be distinct from that of Caucasian women (Borgdorff et al., 2017; Gupta, Paul & Dutta, 2017; Ravel et al., 2011) However, a recent study by Beamer et al. (2017) found no significant difference in the vaginal microbiomes of these two ethnic groups that were free of bacterial vaginosis (BV), *Chlamydia trachomatis*, *Neisseria gonorrhoeae* and *Trichomonas vaginalis* infections. The microbial composition of the vaginal microbiome has been clustered into five structures, referred to as community state types (CSTs) I, II, III, IV and V, based on the relative abundance (RA) of four microaerophilic *Lactobacillus* species (*L. crispatus*, *L. gasseri*, *L. iners* and *L. jensenii*) and a heterogenous group of microbes. CST I is dominated by *L*. *crispatus*, and CST II, III and V by *L. gasseri*, *L. iners* and *L. jensenii*, respectively. CST IV, considered a dysbiotic state, is *Lactobacillus* deficient and consists of a polymicrobial group of strict obligate and facultative anaerobes (such as *Prevotella*, *Gardnerella*, *Atopobium*, *Aerococcus, Anaerococcus*, *Dialister*, *Finegoldia, Peptoniphilus*, *Parvimonas*, *Megasphaera*, *Sneathia*, *Eggerthella*, *Veillonella, Gemella, Peptostreptococcus, Bifidobacterium, and Corynebacterium* along with rarer taxa) (Fettweis et al., 2014; Ravel et al., 2011). Recognised among this polymicrobial group are bacteria such as *Campylobacter*, *Enterococcus*, *Escherichia*, *Haemophilus, Mycoplasma*, *Shigella, Staphylococcus* and *Streptococcus* which have previously been referred to as “pathobionts”, symbionts implicated in host pathology due to chronic uncontrolled inflammation (Chow, Tang & Mazmanian, 2011; van de Wijgert et al., 2020). However, since there is no evidence of causality and all human symbionts are potentially pathogenic (Jochum & Stecher, 2020), within the context of the vaginal microbiome we shall refer to these organisms as “taxa with high pathogenic potential” (THPP). Several THPP and other anaerobes reside in *Prevotella–*enhanced *G. vaginalis* biofilms (dynamic, highly organized adherent bacterial communities self-encased in an extracellular polysaccharide (EPS) that forms a matrix) (Patterson et al., 2007). The vaginal biofilm allows *G. vaginalis* to thrive in the presence of high concentrations of lactic acid, hydrogen peroxide, bactericidins and antibiotics produced by lactobacilli and other competing micro-organisms (Machado & Cerca, 2015; Castro, Machado & Cerca, 2019). Like *Prevotella* spp. and *G*. *vaginalis*., some *Lactobacillus* species (such as *L*. *jensenii*) have been shown to form vaginal biofilms *in vivo* (Ventolini, 2015). Disruption of microbiota biofilms can lead to the release of THPP and other associated bacteria into the local milieu (Buret et al., 2019).

With widespread high-throughput metagenomic sequencing and characterization of vaginal microbiomes across global hemispheres, CSTs dominated by genera other than *Lactobacillus* have been reported (*e.g*., *Gardnerella vaginalis*, *Prevotella*, *Bifidobacterium* and THPP, such as *Staphylococcus* and *Streptococcus*) (Anukam et al., 2019; Borgdorff et al., 2017). In addition, the findings of recent studies involving healthy black African women (Nigerian, Kenyan, Rwandan and South African) who were found to have *Lactobacillus*-dominant vaginal microbiomes (Anukam et al., 2019; Jespers et al., 2017*)*, are at variance with the results of scientific research emanating from the U. S. A and the Netherlands where the vaginal microbiomes of women of African ancestry (African American, African Surinamese and African Ghanaian) were predominantly of CST IV (Borgdorff et al., 2017; Fettweis et al., 2014; Ravel et al., 2011). In this study, we sought to examine microbial vaginal composition and structure across varied ethnicities by characterizing and analysing the vaginal microbiome of Afro-Caribbean, African American, African Kenyan, Asian Indonesian and Caucasian German women. Additionally, the theoretical relationships between variation in relative abundance of THPP (singly and combined), facultative (*Lactobacillus*) and obligate anaerobes (*Gardnerella* and *Prevotella*) were also assessed.

## Materials and Methods

### Genomic DNA extraction and 16S rDNA V4 sequencing by MiSeq illumina

Cervicovaginal sampling from 18 Afro-Caribbean female participants, gDNA extraction using InstaGene™ Matrix (Bio-Rad Laboratories, Hercules, CA, USA) and amplification of the V4 region of 16S rRNA gene [using custom adaptor ligated primers 515F 5′- GTGCCAGCMGCCGCGGTAA-3′ and 806R 5′- GGACTACHVGGGTWTCTAAT-3′ (Caporaso et al., 2011; Moustafa et al., 2018)] were conducted as previously described (Roachford et al., 2021). Illumina sequencing was performed at J. Craig Venter Institute (JCVI), La Jolla, California, USA. This research was approved (IRB# 170710-B) by the Research Ethics Committee (REC) and Institutional Review Board (IRB) of The University of the West Indies, Cave Hill Campus, Barbados.

### Vaginal microbiome 16S rRNA V4 datasets

Overall, this study involved the analysis of vaginal microbiome 16S rRNA V4 datasets from a total of 151 women (regardless of health status) of five different ethnicities. Of the 151 DNA datasets, 133 were from published data and 18 from the experimental DNA, representing women of five different ethnicities. Datasets were sub-sampled and downloaded from the European Nucleotide Archive (ENA): African American (AA) (PRJNA422447, *N* = 28), African Kenyan (AK) (PRJNA516684, *N* = 31), Asian Indonesian (AI) (PRJEB34323, *N* = 36) and Caucasian German (CG) (PRJEB17077, *N* = 38). With respect to the CG dataset, the V4 region was trimmed from the V3–V4 hypervariable sequence using Trimmomatic v0.39 (Bolger, Lohse & Usadel, 2014). DNA datasets from the 151 women were grouped and concatenated based on ethnicity, for downstream analyses. The cervicovaginal microbiome of the Afro-Caribbean (AC) women (*N* = 18) were characterized and analyzed along with those of AA, AK, AI and CG women.

### Bioinformatics and statistical analysis of 16S rRNA V4 data

Quantitative Insights Into Microbial Ecology (QIIME 2, version 2019.10) was used to analyze the 16S rRNA V4 hypervariable region of the datasets following importation into a QIIME 2 artifact. DADA2 plugin was utilized to generate an amplicon sequence variant (ASV) feature table after quality control (sequence correction, de-replication, low-quality filtering and chimeric sequence removal). Taxonomic assignment to ASVs at 97% sequence similarity was achieved using a Naive-Bayes classifier trained on the V4 region of SILVA_138_SSURef_NR99 database. The feature table was rarefied to a sampling depth of 80,000 with q2-diversity core-metrics-phylogenetic plugin. Alpha and Beta diversities were determined using Shannon’s diversity index and unweighted UniFrac distance metric, respectively. Taxonomic barplots, boxplots and three-dimensional principal coordinates analysis (3D-PCoA) plots were generated in QIIME 2 (Caporaso et al., 2010) using taxa barplot, diversity alpha-group-significance and emperor plot plugins, respectively. Permutational multivariate analysis of variance (PERMANOVA) and Kruskal–Wallis tests also implemented in QIIME 2 were used to assess whether there were significant microbial composition differences among ethnic groups. To identify the bacterial taxa that accounted for meaningful variance in the microbial composition among the various ethnicities, linear discriminant analysis (LDA) effect size (LEfSe) in a python 2.7 conda environment was utilized. In the analysis, a non-parametric factorial Kruskal–Wallis sum-rank test within LEfSe identified the differentially abundant taxa and LDA quantified their effect on microbial composition variance (Segata et al., 2011). A LEfSe barplot was produced where horizontal bars represented the effect size for the differentiating feature (bacterial taxa). An LDA effect size >2 and a *p*-value < 0.05 were used as cutoffs. Community state types (CSTs) for vaginal samples were generated by hierarchical clustering using the complete linkage method and visualized as a heatmap using the *hclust* function and ComplexHeatmap package in R version 4.0.3 (R Core Team, 2020). The relationships between strict anaerobes (*Prevotella* and *Gardnerella*), *Lactobacillus* species, THPP and specifically *M*. *hominis* were explored using Pearson’s correlation coefficient (when linear covariation) and Spearman’s rank correlation (where data not normally distributed) tests. Distribution of the variables was checked and visualized using the Shapiro–Wilk normality test and *ggqqplot* function in *ggpubr* R package. Correlation coefficients and plots for associations between bacterial taxa were generated with the *cor.test* and *ggscatter* functions (*ggpubr* R package), respectively.

## Results

### Amplicon sequence characteristics

Samples from the five ethnic groups yielded a total of 8,527,099 amplicon sequences. Filtration of low quality reads and chimeras generated 4,368,987 sequences: Afro-Caribbean (355,080), African American (91,809), African Kenyan (994,995), Asian Indonesian (1,080,047) and Caucasian German (1,847,056) (Table S1). The Amplicon Sequence Variant (ASV) feature table produced 2,521 features with sequence lengths of 250 bp across all five ethnic groups. A total of 663 taxa were identified after sequences were matched against SILVA database using feature-classifier, classify-sklearn, in QIIME 2 (Table S2).

### Cervicovaginal microbiome composition across ethnic groups

**Figure 1:**
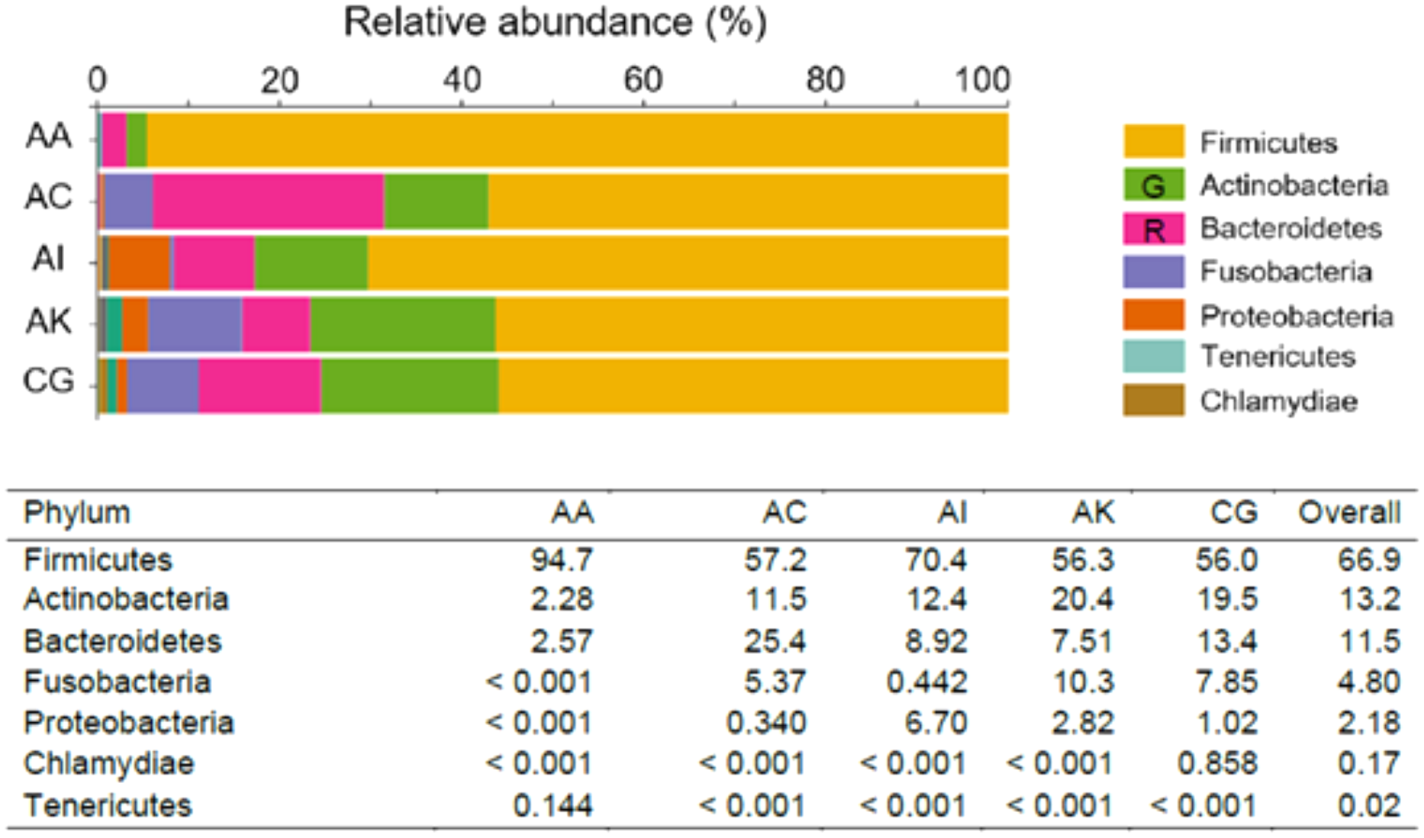
Taxonomic pattern and percentage relative abundance of the top seven phyla within the vaginal microbiomes of the five ethnic groups (AA, African American; AC, Afro-Caribbean; AI, Asian Indonesian; AK, African Kenyan and CG, Caucasian German). G = Green; R = Red.

The most abundant phyla (RA > 0.10%) recognized across the five ethnic groups were Firmicutes (lead by AA, with an RA of 94.7%), followed by Actinobacteria (lead by AK, RA = 20.4%) and Bacteroidetes (predominant in AC, RA = 25.4%). Fusobacteria was of greater abundance in AK (10.3%), whereas Proteobacteria predominated in AI (6.70%). Two other phyla, Tenericutes and Chlamydiae, with RA between 0.10–1.00% were only found in AA (0.144%) and CG (0.858%), respectively (Fig. 1). At the genus level, *Lactobacillus* was the most dominant amongst the five ethnic groups, RA ranged from 26.1% to 91.8% in AC and AA, respectively. Overall, the RA of *Lactobacillus* species was 47.9%. The *Lactobacillus* species identified were *L. crispatus, L. iners, L. gasseri, L. equi, L. mucosae, L. murinus* and *L. salivarius*. The next most abundant genus was *Prevotella* (which consisted of *P. denticola, P. amnii, P. bivia, P. buccalis, P. disiens* and *P. ihumnii* ) accounted for 8.92% of all taxa among the five ethnic groups. It was most abundant among AC women (21.0%) and least among AA women (2.11%). Genera/species identified among the ethnic groups, with RA > 1.00% were *Gardnerella* which consisted *G. vaginalis* (8.40%), *Sneathia* (*S. amnii, S. sanguinegens*; 4.57%), “*Candidatus* Lachnocurva vaginae” (formerly *Shuttleworthia* and BVBA1 (Holm et al., 2020), 3.41%), *Anaerococcus* (*A. lactolyticus, A. prevotii, A. provenciensis, A. vaginalis*; 2.57%), *Megasphaera* (*M. indica*; 2.30%), *Atopobium* (*A. deltae, A. minutum*; 12%), *Corynebacterium* (*C. aurimucosum*, *C. genitalium*, *C. jeikeium*, *C. kroppenstedtii*, *C. durum*, *C. Pyruviciproducens*; 1.16%), *Escherichia–Shigella* (1.14%) and *Enterococcus* (1.14%). Other significant taxa identified within the vaginal microbiomes but with relative abundance <0.10% were *Staphylococcus*, *Veillonella*, *Fastidiosipila*, *Bifidobacterium*, *Peptostreptococcus*, *Peptoniphilus*, *Dialister*, *Porphyromonas*, *Gemella*, *Finegoldia*, *Mobiluncus*, Parvimonas, *Ureaplasma parvum*, *Mycoplasma hominis* and *Moryella indoligenes*. *Chlamydia trachomatis* which was notable among Caucasian German women (0.857%), was absent or less than 0.001% among women of other ethnicities (Tables 1 and S3).

**Table 1:** Relative abundance of top 30 taxa at genus level across five ethnic groups.

	Relative abundance (%) in ethnic groups						
Taxa (genus level)	AA	AC	AI	AK	CG	Overall (%)	Species identified
*Lactobacillus*	91.8	26.1	45.0	34.4	42.4	47.9	*L. crispatus, L. iners, L. gasseri, L. equi, L. mucosae, L. murinus, L. salivarius*
*Gardnerella*	0.884	9.91	6.24	12.1	12.8	8.40	*G. vaginalis*
*Prevotella*	2.11	21.0	5.05	4.52	12.0	8.92	*P. denticola, P. amnii, P. bivia, P. buccalis, P. disiens, P. ihumnii*
*Sneathia*	0.00	5.26	0.001	9.77	7.81	4.57	*S. amnii, S. sanguinegens*
*Megasphaera*	0.001	3.51	0.307	2.45	5.25	2.30	*M. indica*
*Atopobium*	1.36	0.909	0.667	3.34	4.34	2.12	*A. deltae, A. minutum*
*Anaerococcus*	0.094	6.95	2.84	2.79	0.206	2.57	*A. lactolyticus, A. prevotii, A. provenciensis, A. vaginalis*
*Shuttleworthia* (BVBA1)	0.00	15.3	0.00	1.06	0.711	3.41	–
*Corynebacterium*	0.00	0.185	2.14	3.50	0.00	1.16	*C. aurimucosum, C. genitalium, C. jeikeium, C. kroppenstedtii, C. durum, C. Pyruviciproducens*
*Veillonella*	0.00	0.511	0.069	0.902	1.98	0.692	*V. montpellierensis, V. seminalis*
*Fastidiosipila*	0.00	0.620	0.705	1.86	0.758	0.788	Unidenitfied
*Bifidobacterium*	0.00	0.001	0.519	0.151	1.73	0.479	*B. breve, B. avesanii, B. dentium, B. longum*
*Peptoniphilus*	0.148	0.285	2.29	0.704	0.001	0.686	*P. lacrimalis, L. obesi, L. phoceensis*
*Dialister*	0.394	1.58	0.207	1.10	0.775	0.811	*D. pneumosintes*
*Porphyromonas*	0.272	2.08	0.450	0.856	0.480	0.827	*P. asaccharolytica*
*Gemella*	0.00	0.602	0.247	0.173	1.17	0.439	*G. asaccharolytica, G. haemolysans*
*Prevotella 6*	0.001	1.83	1.06	0.360	0.342	0.718	–
*Finegoldia*	0.315	0.001	1.94	0.478	0.001	0.547	*F. magna*
*Mobiluncus*	0.000	0.001	1.53	0.835	0.001	0.474	*M. curtisii*
*Parvimonas*	0.000	0.088	0.550	0.471	0.560	0.334	Unidenitfied
*Moryella*	0.00	0.043	0.572	1.17	0.001	0.357	*M. indoligenes*
*Chlamydia*	0.00	0.00	0.00	0.00	0.857	0.171	*C. trachomatis*
*Streptococcus*	0.001	0.001	2.02	4.98	0.597	1.52	*S. agalactiae, S. anginosus, S. salivarius*
*Escherichia*	0.00	0.00	4.59	1.08	0.000	1.14	Unidenitfied
*Enterococcus*	0.00	0.00	4.02	0.512	0.573	1.02	Unidenitfied
*Peptostreptococcus*	1.64	0.001	2.72	0.184	0.293	0.967	Unidenitfied
*Staphylococcus*	0.001	0.001	2.68	2.05	0.038	0.954	Unidenitfied
*Ureaplasma*	0.144	0.001	0.084	1.43	0.251	0.383	*U. parvum*
*Mycoplasma*	0.00	0.001	0.001	0.310	0.805	0.223	*M. hominis*
Unassigned bacteria	0.00	0.000	0.730	0.797	0.00	0.305	–

**Note:**

AA, African American; AC, Afro-Caribbean; AI, Asian Indonesian; AK, African Kenyan; CG, Caucasian German. *Shuttleworthia* (BVAB1) has been renamed as Candidatus *Lachnocurva vaginae* (Holm et al., 2020).

### Alpha and beta diversity

**Figure 2:**
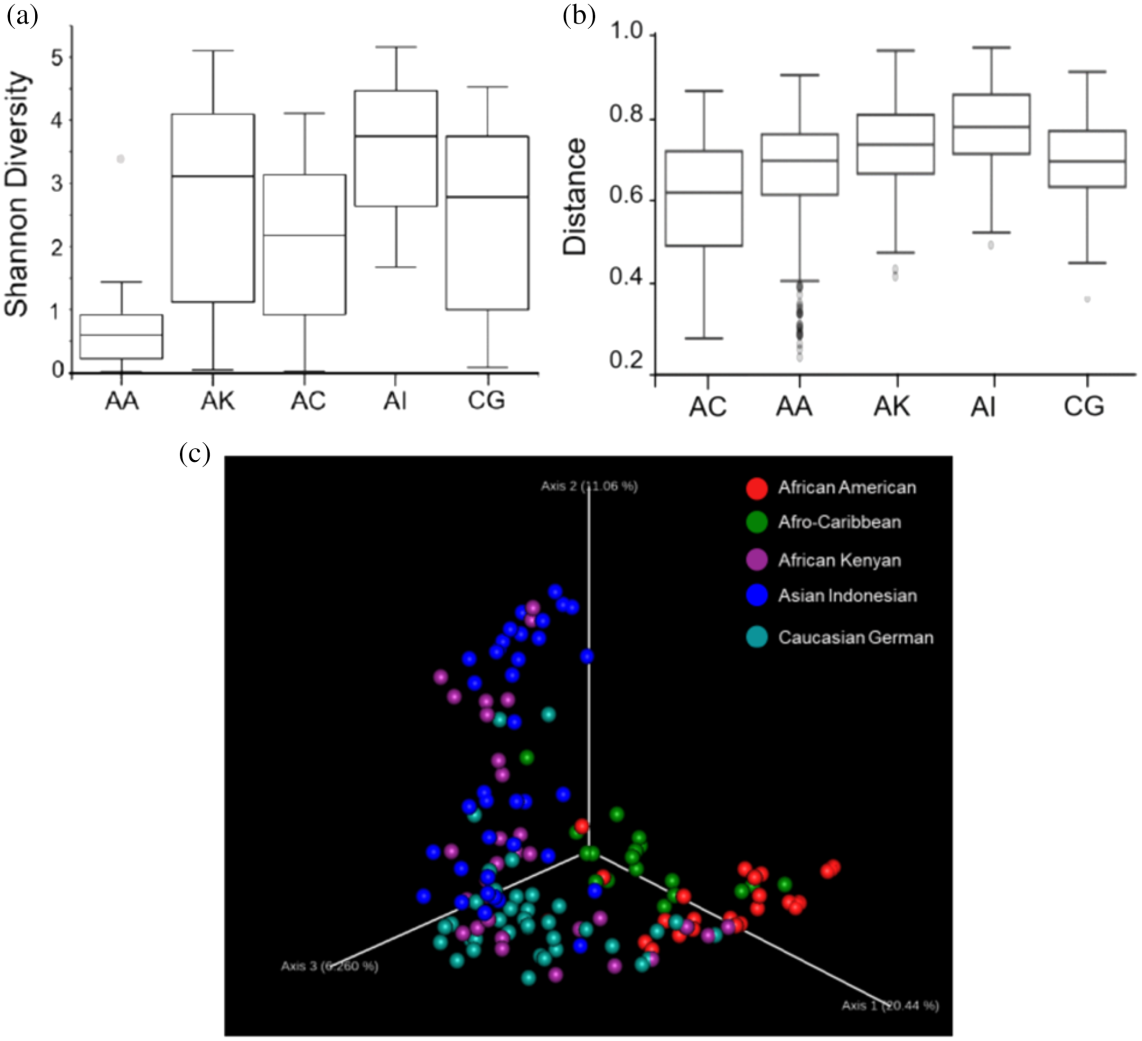
Plots showing species richness and diversity among the five ethnic groups (AA, African American; AC, Afro-Caribbean; AI, Asian Indonesian; AK, African Kenyan and CG, Caucasian German). (A) Boxplot shows distribution of species richness and evenness within the vaginal microbiomes of the ethnic groups using Shannon diversity matrix. (B) Boxplot showing the distribution of unweighted unifrac distances among the five ethnicities. (C) Three-dimensional principal coordinates analysis (3D-PCoA) visualization of pairwise Bray-Curtis distance matrices indicating vaginal bacterial species diversity among the five ethnicities.

**Figure 3:**
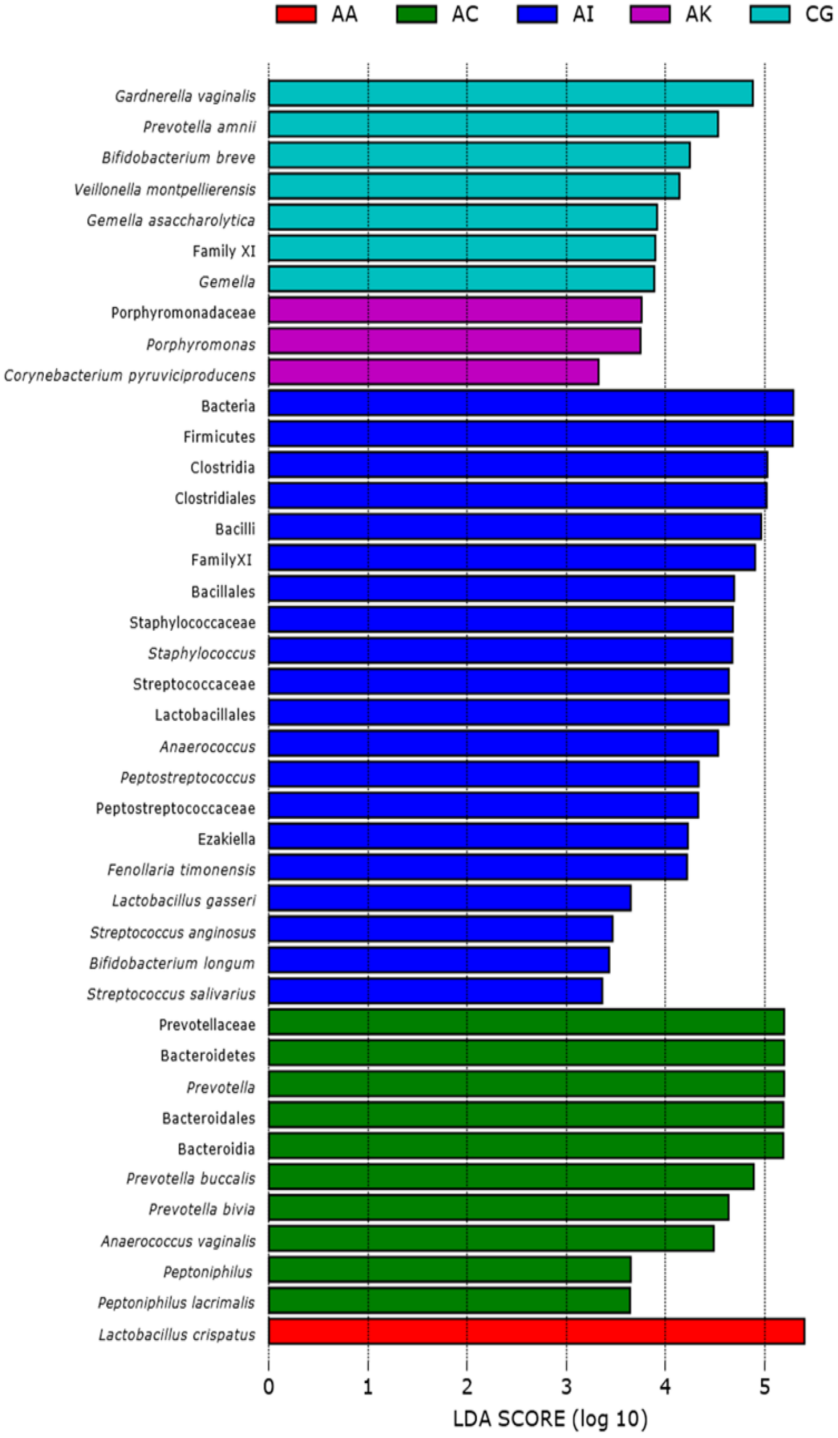
Linear discriminant analysis (*LEfSe* with abs LDA score >2.0) plot showing differentiating bacterial taxa of the vaginal microbiomes of the five ethnic groups (AA, African American; AC, Afro-Caribbean; AI, Asian Indonesian; AK, African Kenyan and CG, Caucasian German). Each horizontal bar represents effect size for the differentiating feature (bacterial taxa).

Based on the Shannon alpha diversity index which assesses bacterial community diversity (amplicon sequence variants) and evenness, there was a statistically significant difference (Kruskal–Wallis (pairwise), *p*-value > 3.59e−11) in species richness within samples across the five ethnic groups. Species richness within the samples from AC women was significantly different to that of AA (*p*-value > 6.50e−04) and AI (*p*-value = 0.0003) women but not significantly different to that of samples from AK and CG women (*p*-value = 0.120 and 0.262, respectively) (Fig. 2A and Table S1). Statistically, species diversity (Beta diversity) as determined by Unweighted UniFrac matrix, was significantly different (PERMANOVA, *p*-value = 0.001) among the five ethnic groups as shown in boxplots (Fig. 2B and Table S1), where a diversity distance of 0 represents identical bacterial composition of the vaginal microbiome and a distance of 1 indicates total dissimilarity. The 151 vaginal samples clustered into five groups as visualized by 3D-PCoA (Unweighted–UniFrac diversity matrix), representing the (Beta) diversity of the vaginal microbiomes of the five ethnic groups (Fig. 2C). To explain the beta diversity among the ethnic groups, linear discriminant analysis (*LEfSe* with abs LDA score > 2.0) was performed. The *LEfSe* plot (Fig. 3) shows the discriminant taxa observed within the vaginal microbiomes of the five ethnic groups. *L. crispatus* was differentially of higher abundance in African Americans. Additionally, the variance in species diversity among AC, AI, AK and CG women could be explained by the differential abundance of *Prevotella* spp., THPP, *Corynebacterium, Porphyromonas* spp., *Gemella* spp. and *G. vaginalis*, respectively.

### Hierarchical clustering analysis of the vaginal samples for all ethnic groups

**Figure 4:**
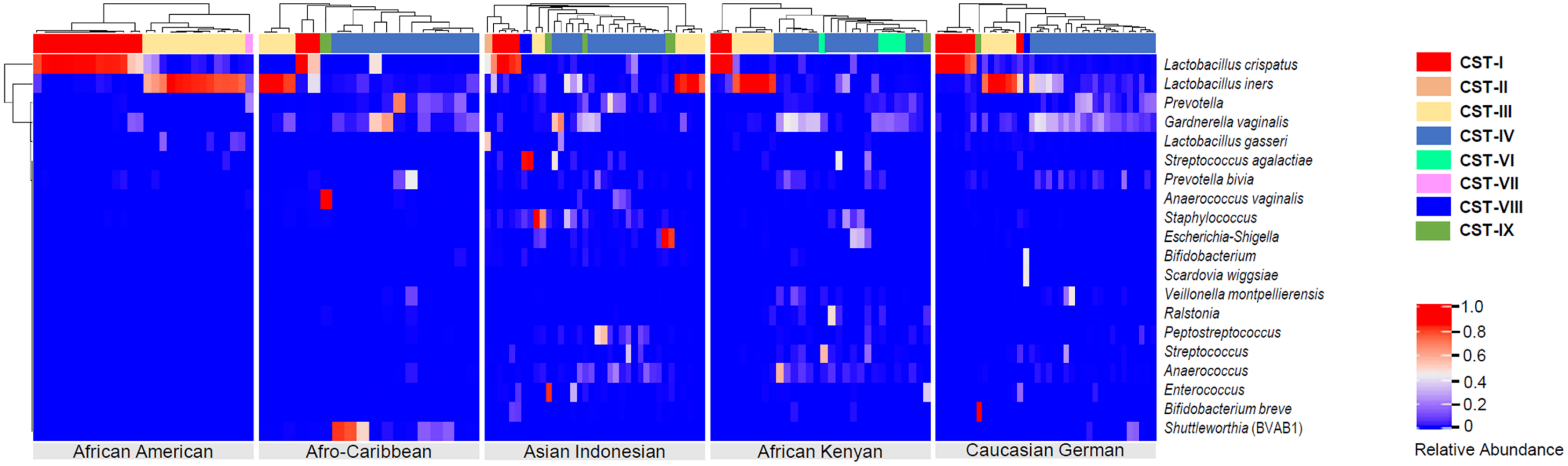
Heatmap displaying the relative abundance of the top 20 bacterial taxa within the vaginal microbiomes of five ethnic groups (African American, Afro-Caribbean, Asian Indonesian, African Kenyan and Caucasian German). Hierarchical clustering of the 151 vaginal samples resulted in eight clusters designated as community state types: CST I–IV and VI–IX.

Hierarchical clustering analysis of the vaginal samples, using the complete linkage method, was performed to find similar clusters within the samples. Eight clusters, representing CSTs, were found and visualized as a heatmap (Fig. 4). An organism was considered to be dominant within each CST where its percentage RA was equal or greater than 50%. Overall, CSTs (Table 2) were predominated by *Lactobacilli* species: *L*. *iners*—dominant clusters (CST III, *N* = 38, 25.2%) and *L*. *crispatus*—dominant clusters (CST I, *N* = 33, 21.8%). There was only one *L*. *gasseri* dominant cluster (CST II) and no *L*. *jensenii*—dominant clusters. CST IV, a polymicrobial community state, was the most common CST *(N* = 56, 37.1%) among all women. Three subtypes of CST IV (CST IV-A, IV-B and IV-C) were identified: CST IV-A consisted of heterogeneous bacteria with *G. vaginalis* in high proportions (but RA < 50%), CST IV-B consisted of heterogeneous bacteria and *Prevotella* (RA ≤ 45%) and CST IV-C which comprised of heterogeneous bacteria along with any predominant microbe other than *Gardnerella* or *Prevotella* (RA < 50%). Predominant bacteria identified within CST IV-C were *Bifidobacterium*, *Corynebacterium*, *Clostridiales* bacterium, *L*. *iners*, *Peptostreptococcus*, *Streptococcus* and *Ureaplasma parvum*. Other clusters identified were: *G*. *vaginalis*—dominant clusters [(CST VI) (*N* = 6, 21.8% of women), present in AC, AI and AK women only], a *Prevotella*—dominant cluster [(CST VII) (*N* = 1, 0.66% of women) found in an AC woman], THPP—dominant clusters [(CST VIII) (*N* = 9, 5.96%), present only in AI and AK women] and CST IX (*N* = 7, 4.64%) which represented a group of clusters dominated by bacteria other than *Lactobacilli*, *G*. *vaginalis*, *Prevotella* and THPP. Clusters within this CST (IX) mainly occurred as singletons (*i.e*., occurring in only one sample) in all ethnic groups except African Americans. Six bacteria dominated in this cluster: *Bifidobacterium breve* (RA = 98%), *Finegoldia* (RA = 71%), *Anaerococcus vaginalis* (RA = 93%), *Anaerococcus* (RA = 62% ), *Ralstonia* (RA = 51%) and *Lachnocurva vaginae* which was dominant in two samples (RA ≥ 80%).

**Table 2:** Cervicovaginal microbiome structures and community state types across five ethnic groups.

**Cervicovaginal microbiome structure**	**Cluster description, relative abundances of taxa (%)**	**Assigned CST**	**AA**	**AC**	**AI**	**AK**	**CG**
***L. crispatus* predominant, RA > 50%**	*L. crispatus—*dominant *> 98.9%*	I	5	1		2	4
	*L. crispatus—*dominant *> 97%, Prevotella bivia > 0.5%*	I	2				
	*L. crispatus—*dominant *> 97%, G. vaginalis > 2.0%*	I					1
	*L. crispatus—*dominant *> 92%, L. iners > 0.5%*	I	5		2	1	
	*L. crispatus—*dominant *> 66%, L. iners > 30%*	I			1		
	*L. crispatus—*dominant *≥ 55%, L. iners 40%*, heterogenous *bacteria*	I		1			
	*L. crispatus—*dominant *> 90%, L. iners > 4%, Corynebacterium* 1 *> 1.0%*	I			1		
	*L. crispatus—*dominant *> 57%, Atopobium >20%, G. vaginalis >14%*	I	2				
	*L. crispatus—*dominant *> 86%, G. vaginalis > 5.0%, Streptococcus agalactiae > 2.5%, Staphylococcus > 1.6%*	I			1		
	*L. crispatus—*dominant *> 86%, L. iners > 3.0%*, heterogenous *bacteria*	I					1
	*L. crispatus—*dominant *> 80%, Bifidobacterium breve > 6.3%, Enterococcus > 5.2%*	I			1		
	*L. crispatus—*dominant *> 80%, Bifidobacterium breve > 6.3%, Streptococcus > 3.1%*	I			1		
	*L. crispatus—*dominant *≥ 80%, G. vaginalis > 8.0%*, heterogenous *bacteria*	I					1
	**Number/percentage of samples CST-I**		**14 (50%)**	**2 (11%)**	**7 (19%)**	**3 (9.7%)**	**7 (18%)**
***L. gasseri* predominant, RA > 50%**							
	*L. gasseri—*dominant 52%, *L. iners* 43%	II			1		
	**Number/percentage of samples CST- II**		0	0	1 (2.8%)	0	0
***L. iners*** **predominant, RA > 50%**							
	*L. iners—*dominant ≥ 98%	III	2	2	1	6	1
	*L. iners—*dominant > 90%, *L. crispatus* > 1.5%	III	3			1	1
	*L. iners—*dominant 60–75%, *L. crispatus* > 24%	III	2				
	*L. iners—*dominant > 80%, *L. gasseri* 2.8–17%	III	4				
	*L. iners—*dominant *≥ 80%*, heterogenous bacteria	III			3		1
	*L. iners—*dominant *72%*, unassigned bacteria *25%*, heterogenous bacteria	III			1		
	*L. iners—*dominant > 80%, *G. vaginalis 4.0%—* ≥ 10%	III		1			4
	*L. iners—*dominant >55*G. vaginalis* > 25% *Atopobium*	III	2				1
	*L. iners > 50%* THPP *= Staphylococcus, Streptococcus, Enterococcus*	III				1	1
	**Number/percentage of samples CST- III**		**13 (46%)**	**3 (17%)**	**5 (14%)**	**8 (26%)**	**9 (24%)**
**Polymicrobial microbiome with *G. vaginalis* most dominant but with a RA < 50%**							
	Heterogenous bacteria, *G. vaginalis 44%*, *Atopobium 32%, Veillonella montpellierensis 11%*	IV-A				1	
	Heterogenous bacteria, *G. vaginalis* 28–32% and *Prevotella bivia* 11–15%	IV-A			2	1	
	Heterogenous bacteria, *G. vaginalis 34% Prevotella amnii 6.4%*	IV-A			1		
	Heterogenous bacteria, *G. vaginalis 36% Prevotella amnii 25–34%, Atopobium 17–23%*	IV-A				2	
	Heterogenous bacteria, *G. vaginalis 25–45% L. iners 37–44%*	IV-A			1	1	4
	Heterogenous bacteria, *G. vaginalis > Prevotella, Atopobium, Streptococcus, Veillonella, Anaerococcus*	IV-A				4	2
	Heterogenous bacteria, *G. vaginalis > Prevotella, Shuttleworthia, Megasphaera, Sneathia, L. iners*	IV-A				1	4
	**Number/percentage of samples CST- IV-A**		**0**	**0**	**4 (11%)**	**10 (32%)**	**10 (26%)**
**Polymicrobial microbiome with *Prevotella* species most dominant but with a RA < 50%**							
	Heterogenous bacteria, *Prevotella 18%, low abundance Lactobacilli*	IV-B			3		
	Heterogenous bacteria, *Prevotella > 36%, No Gardnerella*	IV-B		2			
	Heterogenous bacteria, *Prevotella 16–46%*	IV-B		1	2		
	Heterogenous bacteria, *Prevotella > G. vaginalis, Shuttleworthia, Megasphaera, Sneathia amnii, L. iners*	IV-B		2			6
	Heterogenous bacteria, *Prevotella > G. vaginalis, Gemella, Megasphaera, Sneathia amnii, L. iners, M. hominis*	IV-B		2			4
	**Number/percentage of samples CST- IV-B**		**0**	**7 (39%)**	**5 (14%)**	**0**	**10 (26%)**
**Polymicrobial microbiome with genera other than *Gardnerella* or *Prevotella* most dominant but with a RA < 50%**							
	Heterogenous bacteria, *L. iners 25–45%*				3		
	Heterogenous bacteria, *Corynebacterium 1 27%*	IV-C			1		
	Heterogenous bacteria, *Bifidobacterium 47%, Scardovia wiggsiae 49%*	IV-C					1
	Heterogenous bacteria, *Streptococcus agalactiae -dominant 46%, L. iners 13%, Sneathia spp. 10%*	IV-C			2		
	Heterogenous bacteria, *Ureaplasma parvum* 36%, *Escherichia 33%, Staphylococcus 16%*	IV-C				1	
	Heterogenous bacteria, *Clostridiales* bacterium 34%, *Shuttleworthia* 16%, *Veillonella* 6.6%	IV-C				1	
	Heterogenous bacteria, *Peptostreptococcus* 28%, *Prevotella* 25%, *L. iners* 13%	IV-C	1				
	**Number/percentage of samples CST- IV-C**		**1 (4.0%)**	**0**	**6 (17%)**	**2 (6.4%)**	**1 (2.6%)**
***L. jensenii* predominant, RA > 50%**							
	*L. jensenii—*dominant > *50%*	V	0	0	0	0	0
	**Number/percentage of samples CST- V**		**0**	**0**	**0**	**0**	**0**
***G. vaginalis* predominant, RA > 50%**							
	*G. vaginalis—*dominant *> 53%, L. iners 47%*	VI		1		1	
	*G. vaginalis—*dominant *>60%, Sneathia spp., Shuttleworthia, Megasphaera, Atopobium, Aerococcus*	VI		1		1	
	*G. vaginalis—*dominant *54%, Streptococcus agalactiae 45%*	VI			1		
	*G. vaginalis—*dominant *68%, L. gasseri 22%*	VI			1		
	**Number/percentage of samples CST- VI**		**0**	**2 (11%)**	**2 (5.6%)**	**2 (6.4%)**	**0**
***Prevotella* species dominant, RA > 50%**							
	*Prevotella* sp.*—*dominant *> 70%*, heterogenous bacteria	VII		1			
	**Number/percentage of samples CST- VII**		**0**	**1 (5%)**	**0**	**0**	**0**
**THPP predominant, RA > 50%**							
	*Streptococcus—*dominant *60%, Sneathia sanguinegens 23%, G. vaginalis 9.0%*, heterogenous bacteria	VIII				1	
	*Streptococcus agalactiae-*dominant > 85%, heterogenous bacteria	VIII				2	
	*Peptostreptococcus 54%*, heterogenous bacteria	VIII			1		
	*Enterococcus—*dominant *>80%, Bacilli 15%*	VIII			1		
	*Staphylococcus—*dominant *65–86%, low abundance L. iners, Escherichia*	VIII			2		
	*Escherichia-Shigella—*dominant *88%, low abundance L. iners 5%, Anaerococcus*	VIII			1	1	
	**Number/percentage of samples CST- VIII**		**0**	**0**	**5 (14%)**	**4 (13%)**	**0**
							
**Genera other than *Lactobacillus*, *Gardnerella*, *Prevotella* and THPP that are predominant, RA > 50%**	*Finegoldia*—dominant 71%, heterogenous bacteria	IX			1		
	*Anaerococcus vaginalis -*dominant 93%, *Howardella* 5%	IX		1			
	*Anaerococcus—*dominant 62%, *G. vaginalis 26%, Veillonella montpellierensis 4.7%, Prevotella bivia 2.0%*	IX				1	
	*Shuttleworthia—*dominant ≥80%, *G*. *vaginalis* 2–6.2%, heterogenous bacteria	IX		2			
	*Ralstonia—*dominant *> 51%, Corynebacterium 1 9.0%*, some THPP	IX				1	
	*Bifidobacterium breve—*dominant *> 98%*	IX					1
	**Number/percentage of samples CST- IX**		**0**	**3 (17%)**	**1 (2.8%)**	**2 (6.5%)**	**1 (2.6%)**
	Number of samples in ethnic group (Total No. of samples 151)		28	18	36	31	38

**Note:**

AA, African American; AC, Afro-Caribbean; AI, Asian Indonesian; AK, African Kenyan; CG, Caucasian German; CST = Community State Type. Heterogenous bacteria = low abundance (<7.0%) of various bacteria such as *Sneathia amnii*, *Sneathia sanguinegens*, *Megasphaera*, *Atopobium*, *Clostridiales* bacterium, *Dialister*, *Parvimonas*, *M*. *hominis*, *U*. *parvum*, *Finegoldia*, *Peptoniphilus*, *Mobiluncus*, *Corynebacterium*, *Howardella*, *Porphyromonas*, *Acinetobacter*, *Fusobacterium* among other rare taxa. THPP = *Campylobacter*, *Enterococcus*, *Haemophilus*, *Escherichia*, *Mycoplasma*, *Streptococcus*, *Staphylococcus*, *Ureaplasma*; RA = Relative abundance; *Shuttleworthia* = BVAB1 = Candidatus *Lachnocurva vaginae* (Holm et al., 2020). Numbers and percentages shown in bold represent the total number and percentage of an identified CST in a given ethnic group.

### THPP within the vaginal microbiome

**Figure 5:**
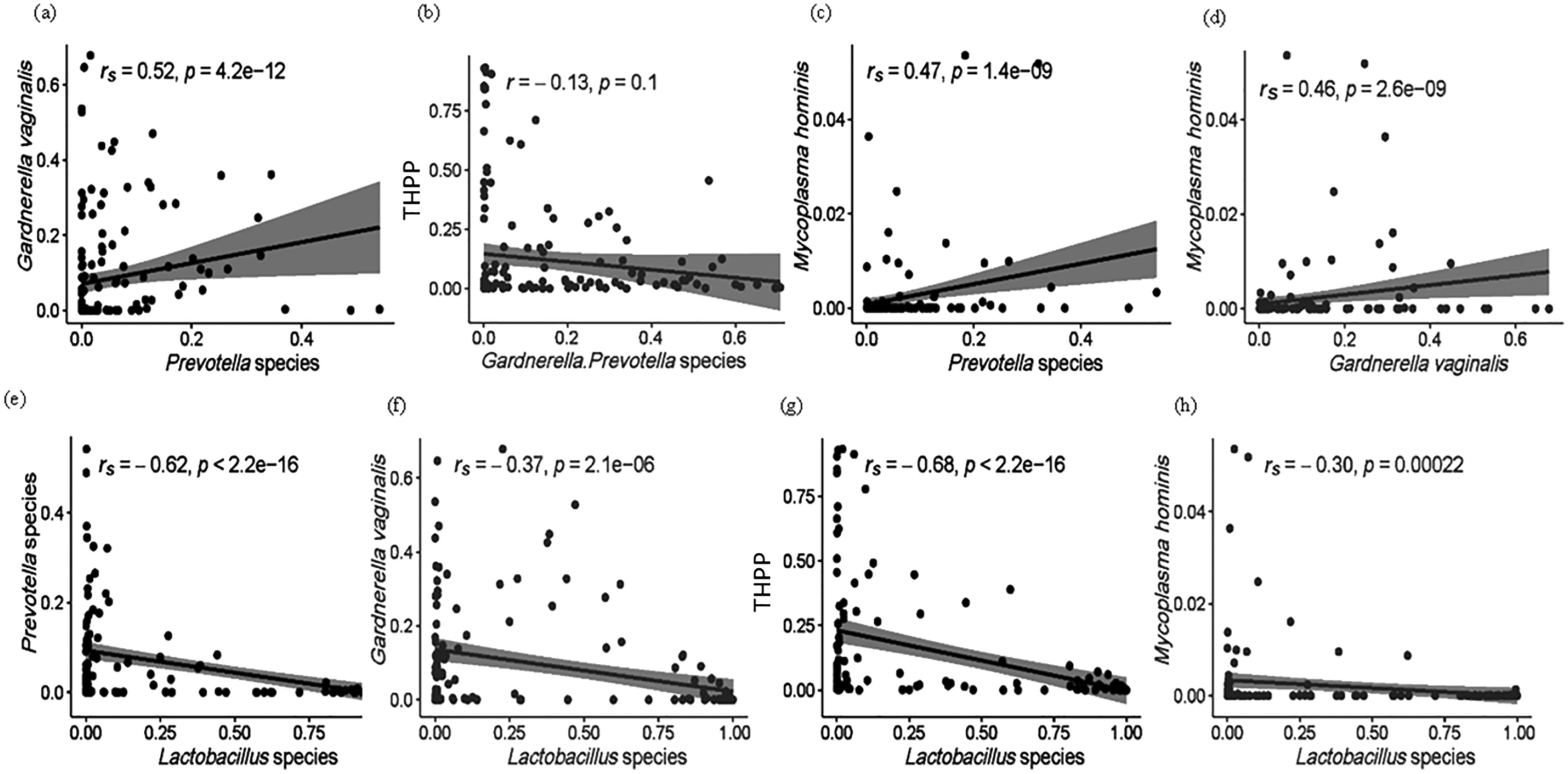
(A–H) Spearman (r_s_) and Pearson (r) correlation plots showing the general theoretical relationship between facultative anaerobes (*Lactobacillus* species), obligate anaerobes (*Gardnerella* and *Prevotella* spp.) and THPP in the vaginal microbiome of the ethnic groups. The strength and direction of the correlation is related to the magnitude and sign of the coefficient value (r): strong correlations, r between ±0.50 and ±1; moderate correlations, r = ±0.30 to ±0.49; weak correlations, r below +0.29. Statistically significant relationships, *p* < 0.05.

Within all 151 samples, 32 THPP taxa at the genus and species taxonomic level were identified (Table S3). Only seven of the 32 THPP taxa identified were among the top 30 bacterial taxa: *Streptococcus* (1.52%), *Escherichia-Shigella* (1.14%), *Enterococcus* (1.02%), *Staphylococcus* (0.954%), *Peptostreptococcus* (0.967%), *U. parvum* (0.383%) and *M. hominis* (0.223%) (Table 1). Species assigned the genus *Streptococcus* were *S. agalactiae*, *S. anginosus* and *S. salivarius*. Two intracellular species, *Chlamydia trachomatis* and *Neisseria gonorrhoeae* which are normally sexually transmitted were identified among the women. *C. trachomatis* was identified in all ethnic groups but with highest RA in CG women. *N. gonorrhoeae*, on the other hand, was only identified in vaginal samples from women of AK and CG ethnicity. *Enterococcus*, *Escherichia–Shigella*, *Peptostreptococcus* and *Staphylococcus* were dominant in the vaginal microbiomes of AI (14%) and AK (13%) women (Table 2). The relationship of THPP (relative abundance levels) with facultative and obligate anaerobes was examined, as well as the association between obligate and facultative anaerobes (Figs. 5 and S1). The relative abundance of THPP showed a strong negative correlation with *Lactobacillus* species (r = −0.68, *p* < 2.2e−16). Generally, there was no significant relationship (r = −0.13, *p* = 0.1) between THPP and the most commonly abundant obligate anaerobes (*Gardnerella*/*Prevotella* relative abundances combined), except for *M*. *hominis*, which correlated positively with *Gardnerella* and *Prevotella* (r_s_ = 0.46, *p* = 2.6e−09 and r_s_ = 0.47, *p* = 1.4e−09, respectively). In contrast, there was a negative correlation (r_s_ = −0.3, *p* = 2.2e−04) between *M*. *hominis* and *Lactobacillus* species (Fig. S1).

## Discussion

### Composition and diversity of the vaginal microbiome among the ethnic groups

In this study, the phylum Firmicutes (which includes the genus *Lactobacillus*) was the most dominant. A *Lactobacillus*-dominant vaginal ecosystem is most common among reproductive age women and is the mainstay of vaginal eubiosis (Ravel et al., 2011). The Actinobacteria phylum was mostly represented by the genera *Gardnerella, Atopobium* and to a lesser extent *Bifidobacterium*, while Bacteroidetes was mostly represented by *Prevotella* (Fig. 1 and Table S2). Firmicutes, Bacteroidetes, Actinobacteria, Proteobacteria and Fusobacteria are the dominant gut microbial phyla (Rinninella et al., 2019) and indeed the most likely origin of the vaginal microbiome (El Aila et al., 2011; Okoli et al., 2019). *Lactobacillus* occurred more than two-fold in AA women (91.8%) compared to women of European (German, 42.4%) and Asian (Indonesian, 45.0%) ancestries. It was also more prevalent in AA women than in other Black women (AC and AK, 26.1% and 34.4%, respectively). This contrasts with other research studies where AA women are expected to have a more diverse microbial composition than Caucasian women (Borgdorff et al., 2017; Fettweis et al., 2014; Ravel et al., 2011). However, the high relative abundance of lactobacilli in the AA cohort is in accordance with studies involving Nigerian and South African women (Anukam et al., 2019; Pendharkar et al., 2013). The observed deviation in microbial composition for AK women might be due to the occurrence of BV (Nugent scores > 7) in this group.

Of the *Lactobacillus* species, *L*. *crispatus* (21.9%) and *L*. *iners* (25.2%) were the two most dominant and were present in all ethnic groups. Other prevalent bacterial taxa with mean relative abundance >1% among the ethnic groups were: *Prevotella* (8.92%), *Gardnerella* (8.40%), *Sneathia* (4.57%), Candidatus *Lachnocurva vaginae* (*Shuttleworthia*) (3.41%), *Anaerococcus* (2.57%) *Megasphaera* (2.30%) and *Atopobium* (2.12%). *Prevotella* abundance was highest among AC women (21.0%) compared to other ethnic groups where the mean relative abundance ranged from 2.11% (AA) to 12.0% (CG). *Gardnerella* was of low abundance in AA women (0.884%) compared to the other ethnic groups where the level ranged from 6.24% (AI) to 12.0% (CG). The high proportions of *Prevotella* and *Gardnerella* in all ethnic groups except for the AA women is indicative of a vaginal dysbiotic state and may further explain why lactobacilli was of lower relative abundance in these ethnic groups. *Prevotella* has been reported to enhance *Gardnerella* biofilm formation in BV which incorporates a number of facultative anaerobes (Castro, Machado & Cerca, 2019; Castro et al., 2021; Machado & Cerca, 2015; Patterson et al., 2010). Additionally, the sexually transmitted bacteria *C. trachomatis* identified at a more significant level in CG women could also have played a role in shifting the bacterial composition in this European group away from the expected *Lactobacillus*-dominated vaginal microbiome (Ziklo et al., 2018).

Overall, vaginal microbial community richness and evenness varied significantly across ethnicities (Kruskal–Wallis, *p* = 3.6e−11), giving the following rank: AI > AK *>* CG *>* AC *>* AA (Fig. 2A and Table S4). Similarly, beta (species) diversity was statistically significant (PERMANOVA, *p* = 0.001) among the ethnic groups (Fig. 2B and Table S4). Beta diversity could be explained by a number of differentiating bacterial taxa as determined by linear discriminant analysis: AA women by the higher abundance of *L. crispatus*, AC women by the predominant *Prevotella* spp., AI women mainly by THPP (such as *Staphylococcus*, *Streptococcus* and *Peptostreptococcus*), AK women by *Porphyromonas* and *Corynebacterium*, and CG women by *Bifidobacterium*, *Veillonella* and *Gamella* (Fig. 3). At the individual level, the vaginal bacterial composition for each woman in this study represented a snapshot of a unique transient microbial signature. Microbial signatures vary temporally with changes in intrinsic (*e.g*., menstrual cycle) and extrinsic factors (*e.g*., sexual activity and antibiotic therapy) (Gajer et al., 2012). There was no definable core microbiome or singular vaginal microbiome signature identified at the species level among all women. This is perhaps a consequence of the involvement of a plethora of confounding variables (such as menstrual cycle phase and hormonal levels, age, parity, marital status, coitus, body mass index [BMI], diet, culture, *etc*.). The microbial composition of the vaginal microbiome is also reportedly influenced in part by factors such as geography, diet, culture and host genetics (Gupta, Paul & Dutta, 2017; Si et al., 2017) which are all distinctive components of ethnicity. Classification of the vaginal bacterial composition into a number of well-defined structures (Ravel et al., 2011) and subtypes, especially with respect to non-*Lactobacillus* CSTs, can serve as an alternative to a core microbiome in understanding the relationship between vaginal microbiome and vaginal health.

### Structure of the vaginal microbiome in various ethnic groups

Vaginal bacterial communities among the ethnic groups clustered into eight CSTs (Table 2). Of the original community state types (CST I–V) (Ravel et al., 2011), CST V (*L. jensenii*) was not identified and CST II (*L*. *gasseri*) was only present in 0.66% of the women. The rarity of CST II and V in the vaginal microbiome of women in this study is in keeping with other studies *(*Chee, Chew & Than, 2020; Doyle et al., 2018). CST I (*L. crispatus*) and CST III (*L. iners*) were the most prevalent *Lactobacillus*—dominant CSTs, 21.9% and 25.2%, respectively. CST I tends to be associated with vaginal microbiota eubiosis (healthy vaginal state) while CST III (and CST IV) with dysbiosis, as for example, in BV (Lehtoranta et al., 2020; Ravel et al., 2011). In this study, CST IV clustered into three subtypes (CST IV–A, IV–B and IV–C) where CST IV–A represented a polymicrobial vaginal microbiome with *G*. *vaginalis* being most dominant but having a relative abundance less than 50%. CST IV–B was also polymicrobial with high proportions of *Prevotella* spp. (RA < 50%). CST IV–C also represented a polymicrobial state with genera other than *Gardnerella* or *Prevotella* being more dominant (RA < 50%). This subtype (CST IV–C) resembled subtypes CST–IVA and IV–B defined in earlier studies by Gajer et al. (2012) and Romero et al. (2014), where CST IV–A was differentiated based on higher abundance of *Peptoniphilus* spp., *Anaerococcus* spp., *Corynebacterium* spp., and *Finegoldia* spp., with respect to *Lactobacillus* species, while CST IV–B was defined by higher abundance of *Atopobium* spp., in addition to *Parvimonas* spp., *Sneathia* spp., *Prevotella* and *Gardnerella*. A *Gardnerella*—dominant CST, classified here as CST VI, has also been identified in African Malawian, African Ghanaian and African Surinamese women (Borgdorff et al., 2017; Doyle et al., 2018) as well as in a group of Caucasian Italian women (De Seta et al., 2019). We also identified a *Prevotella*—dominant vaginal microbiome which was classified as CST VII. CST VIII was differentiated by a high relative abundance (>50%) of a THPP (*Enterococcus*, *Escherichia-Shigella*, *Streptococcus*, *Staphylococcus*, *Peptostreptococcus* species). CST VIII in this study resembles aerobic vaginitis (AV) described as an inflammatory vaginal microbial dysbiotic state predominated by enteric commensals (*e.g*., *Enterococcus* and *Escherichia–Shigella*) or pathogens (Donders, Bellen & Rezeberga, 2011). Finally, CST IX was described here as a CST dominated by any microbe other than *Lactobacillus*, *Gardnerella* or *Prevotella* species with a relative abundance less than 50%. CST IX included a *Bifidobacterium breve*—dominant (RA > 98%) vaginal microbiome in one of the CG women. This distinct CST has also been identified in Italian, Turkish and Moroccan women (Borgdorff et al., 2017; De Seta et al., 2019). Bifidobacteria (inclusive of *B*. *breve* and *B*. *longum*) isolated from vaginal samples have been shown to inhibit pathogens (such as *Staphylococcus aureus*, *Enterococcus faecalis*, *Pseudomonas aeruginosa* and *Escherichia coli*) *in vitro* (Korshunov et al., 1999). *Bifidobacterium* spp. are lactic acid–producing bacteria and as such it is likely, theoretically, that this CST emerged in the presence of a *Lactobacillus*—deficient vaginal environment to guard against the proliferation of pathogenic anaerobes. This concept is supported, in part, by a study involving Thai women where researchers found vaginal *Bifidobacterium* spp. (*B*. *bifidum*, *B*. *longum*, *B*. *breve* and *B*. *dentium*) to be significantly higher in women with BV (Amsel’s criteria) (33.3%) compared to healthy women (11.7%) (Utto et al., 2017). In a limited Iranian study, high concentrations of *Bifidobacterium* spp. and *Lactobacillus* spp., as determined by qPCR assay, were found among healthy women (Bakhshi et al., 2019) suggesting a potential positive role of *Bifidobacterium* in vaginal health. In our study, decreasing proportions (% RA) of *B*. *breve* and *B*. *longum*, were not associated with a significant decrease in the mean RA of *Lactobacillus* spp. (r = −0.76, *p* = 0.35; 95% confidence interval (CI) [−0.23 to 0.084] and r = −0.13, *p* = 0.10; 95% CI [−0.29 to 0.056], respectively) indicating that the RA of *Bifidobacterium* is independent of *Lactobacillus* species and is probably influenced by other changes in the local environment. Vaginal microbiome structures (*i.e*., CSTs) exhibit different levels of stability and can shift temporally from one structure to a next depending on the presence of local or extragenital stressors (Ahrens et al., 2020; Gajer et al., 2012).

### Correlations between THPP, *Lactobacillus* spp., *G*. *vaginalis* and *Prevotella*

The total relative abundance of THPP (with RA > 0.90%) among the five ethnic groups was 5.24%. These were *Streptococcus* (1.52%), *Escherichia–Shigella* (1.14%), *Enterococcus* (1.02%), *Staphylococcus* (0.954%), *Peptostreptococcus* (0.967%), *U. parvum* (0.383%) and *M. hominis* (0.223%). When dominant, their relative abundance ranged from 54% to 88%. All THPP, except the Mollicutes (*Ureaplasma* and *Mycoplasma*), were predominant in 14% of Asian Indonesian women and 13% of African Kenyan women. THPP are of medical importance because they are associated with chronic inflammation (Chow, Tang & Mazmanian, 2011; Jochum & Stecher, 2020) and autoimmune disorders, especially *Mycoplasma* (Roachford, Nelson & Mohapatra, 2017, 2019; Sherbet, 2009). As is known, *Lactobacillus* spp. inhibit the growth of THPP, pathogens and other competitive obligate anaerobes in healthy vaginal ecosystems. It is likely that THPP survive and persist within the vagina due to their integration into protective biofilms formed by *G*. *vaginalis* and *Prevotella* (Patterson et al., 2007). Correlations between THPP and the most common facultative and obligate anaerobes (*Lactobacillus* spp., *G*. *vaginalis* and *Prevotella*) were examined (Figs. 5 and S1). In this study, the relative abundance of both *Gardnerella* and *Prevotella* decreased as the relative proportions of *Lactobacillus* spp. increased (r_s_ = −0.7, *p* = 2.1e−06 and r_s_ = −0.62, *p* < 2.2e−16, respectively) in accordance with a longitudinal study by Gajer et al. (2012) where CST III was shown to shift to CST IV–B, and more recently in a data meta-analysis by van de Wijgert et al. (2020) in which the relative abundance of lactobacilli and BV—anaerobes showed a strong negative correlation. As anticipated, we found a significantly moderate positive correlation (r_*s*_ = 0.52, *p* = 4.2e−12) between the obligate anaerobes *Prevotella* and *Gardnerella*. *P. bivia*, when incorporated into *G*. *vaginalis* biofilms, upregulates the expression of a *G*. *vaginalis* biofilm maintenance gene (HMPREF0424_0821) (Castro et al., 2021), in turn *G*. *vaginalis* enhances biofilm formation by *P. bivia* (Machado, Jefferson & Cerca, 2013). There were moderate positive correlations between *M*. *hominis* and *Gardnerella* (r = 0.46, *p* = 2.6e−09) and *Prevotella* (r = 0.47, *p* = 1.4e−09). *Prevotella*-enhanced *Gardnerella* biofilms are known to harbour THPP such as *Mycoplasma*.

### Limitations of study

In this study with a small sampling size (*N* = 151), the potential effects of confounders (such as age disparities, sociodemographic characteristics, menstrual status, sexual activity *etc*.) on microbial composition were not analysed due to unavailability of data for subsamples from the various ethnic groups. Further, different sampling techniques were utilized and not all women were screened for STIs and BV (*via* Amsel’s or Nugent’s criteria) in some groups by the associated researchers. These factors negatively impact any meaningful interpretation of inter-ethnic comparative analyses of the various vaginal microbiomes. In addition, parametric (*e.g*., ANOVA) and correlation (*e.g*., Spearman’s rank correlation) statistical analyses between two or more features within the vaginal microbiome must be interpreted with caution as these tests are not optimised for microbiome datasets which are high-dimensional (consisting of the co-occurrence of thousands of microbes that influence each other) (*Knight et al., 2018)*. Nonetheless, the strength of this study lies in the use of a more diverse group of women of different ethnicities spanning the Western to Eastern global hemispheres to give a more comprehensive and descriptive assessment of the polymicrobial community state type and subtypes.

## Conclusions

In summary, this study demonstrated that the microbial composition and structure of vaginal microbiomes differed among various ethnicities. However, the high proportion of *Lactobacillus*-dominated vaginal microbiomes in the African American ethnic group seemed to suggest that CST IV might not be the norm in black women as has been posited in some studies. We also found that vaginal microbial structures were not limited to the *Lactobacillus* species (*L*. *crispatus*, *L*. *gasseri*, *L. iners* and *L*. *jensenii*) initially used to classify community state types. In addition, non-*Lactobacillus*-dominated vaginal microbiomes were prevalent and clustered into several CSTs and subtypes resembling bacterial vaginosis and aerobic vaginosis. THPP negatively correlated with *Lactobacillus* species but not with the obligate anaerobes (*Prevotella*/*Gardnerella*), possibly as a consequence of their differential integration into *Prevotella*-enhanced *Gardnerella* biofilms. Larger scale metagenomic studies across diverse ethnicities involving quantitative measurements of bacterial concentrations and visualization of THPP in vaginal biofilms are required to better understand the relationship between CSTs and ethnicity, as well as the association of THPP with other anaerobes within the vaginal microbiome.

## Supplemental Information

10.7717/peerj.14449/supp-1 Supplemental Information 1 Correlation plots. Correlation plots. Spearman (r_s_) and Pearson (r) correlation plots show the theoretical relationship between facultative anaerobes (*Lactobacillus* species), obligate anaerobes (*Gardnerella* and *Prevotella* spp.) and pathobionts in the vaginal microbiome of the ethnic groups.

10.7717/peerj.14449/supp-2 Supplemental Information 2 Sequence characteristics.

10.7717/peerj.14449/supp-3 Supplemental Information 3 Raw abundance of Amplicon Sequence Variant (ASV) taxa identified across 151 women.

10.7717/peerj.14449/supp-4 Supplemental Information 4 Relative abundance of pathobionts in each vaginal sample from all ethnic groups.

10.7717/peerj.14449/supp-5 Supplemental Information 5 Alpha and beta diversity statistics.

## Additional Information and Declarations

### Data Availability

The following information was supplied regarding data availability: The nucleotide sequence data for 16S rRNA V4 are available in the European Nucleotide Archive (ENA), PRJEB34744; MGnify at EMBL-EBI, MGYS00005125; and MG-RAST, mgp89180.
