# Supplementary figures and images for: Insights into the vaginal microbiome in a diverse group of women of African, Asian and European ancestries
Source: PeerJ. 2022 Nov 29;10:e14449. doi: 10.7717/peerj.14449 (PMC9744153; doi:10.7717/peerj.14449)

**Supplementary Figure S1.** Correlation plots

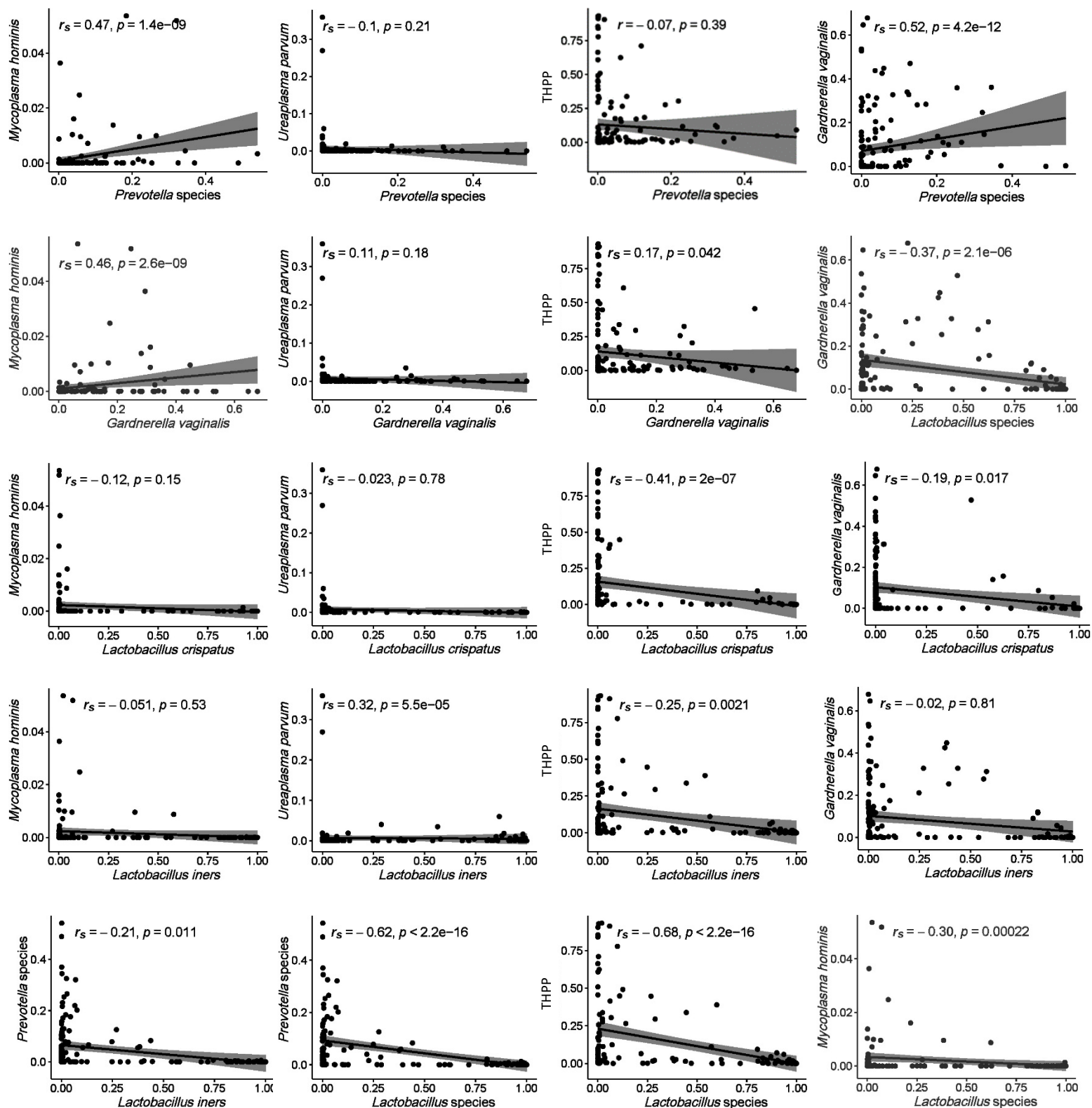

Supplement: Supplemental Information 1 — Correlation plots. Spearman (rs) and Pearson (r) correlation plots show the theoretical relationship between facultative anaerobes (Lactobacillus species), obligate anaerobes (Gardnerella and Prevotella spp.) and pathobionts in the vaginal microbiome of the ethnic groups. [file peerj-10-14449-s001.pdf]
