# Supplementary material for: Insights into the vaginal microbiome in a diverse group of women of African, Asian and European ancestries
Source: PeerJ. 2022 Nov 29;10:e14449. doi: 10.7717/peerj.14449 (PMC9744153; doi:10.7717/peerj.14449)
Supplement: Supplemental Information 5 [file peerj-10-14449-s005.docx]

Supplementary Table S4. Alpha and beta diversity plots and statistics

Alpha Diversity Boxplot - Shannon diversity


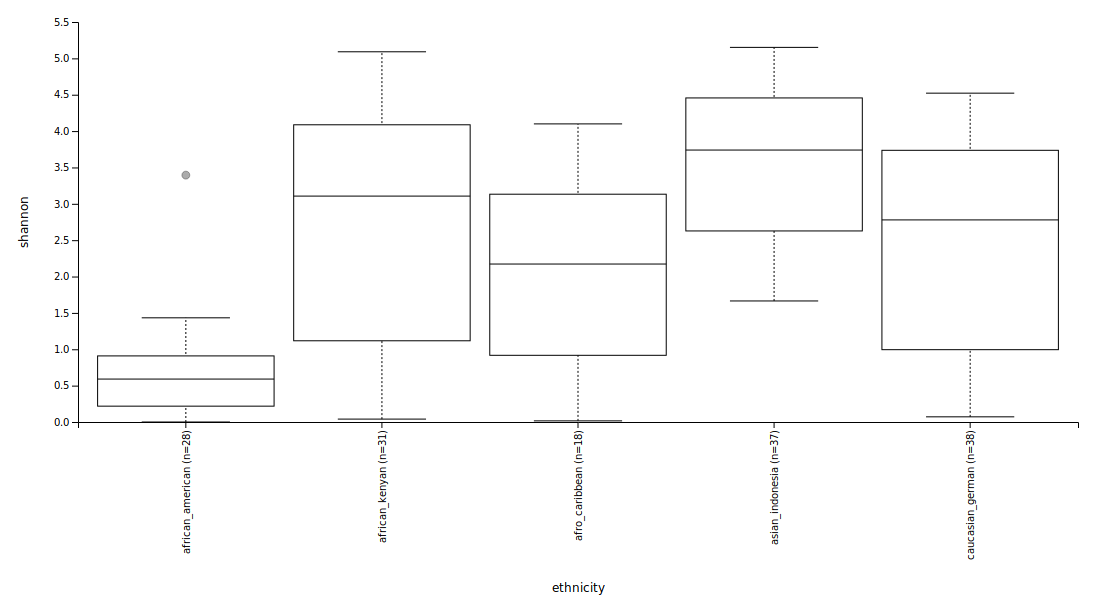


Kruskal-Wallis (pairwise) statistics

| Group 1 | Group 2 | H | p-value | q-value |
| --- | --- | --- | --- | --- |
| African American (n=28) | African Kenyan (n=31) | 23.447235 | 1.283871e-06 | 6.419356e-06 |
| African American (n=28) | Afro-Caribbean (n=18) | 11.627997 | 6.496649e-04 | 1.299330e-03 |
| African American (n=28) | Asian Indonesian (n=37) | 44.403124 | 6.496649e-04 | 2.672603e-10 |
| African American (n=28) | Caucasian German (n=38) | 21.937100 | 2.817343e-06 | 9.391143e-06 |
|  |  |  |  |  |
| African Kenyan (n=31) | Afro-Caribbean (n=18) | 2.419355 | 0.1198442 | 0.1498053 |
| African Kenyan (n=31) | Asian Indonesian (n=37) | 4.939957 | 0.0262426 | 0.0374896 |
| African Kenyan (n=31) | Caucasian German (n=38) | 0.506573 | 0.4766262 | 0.476626 |
|  |  |  |  |  |
| Afro-Caribbean (n=18) | Asian Indonesian (n=37) | 12.999035 | 0.0003116 | 0.000779 |
| Afro-Caribbean (n=18) | Caucasian German (n=38) | 1.2606956 | 0.261519 | 0.290577 |
|  |  |  |  |  |
| Asian Indonesian (n=37) | Caucasian German (n=38) | 10.310212 | 0.0013229 | 0.002205 |

Kruskal-Wallis (all groups): H= 54.79337 p-value = 3.58952e-11

Beta diversity ethnic groups - Unweighted unifrac


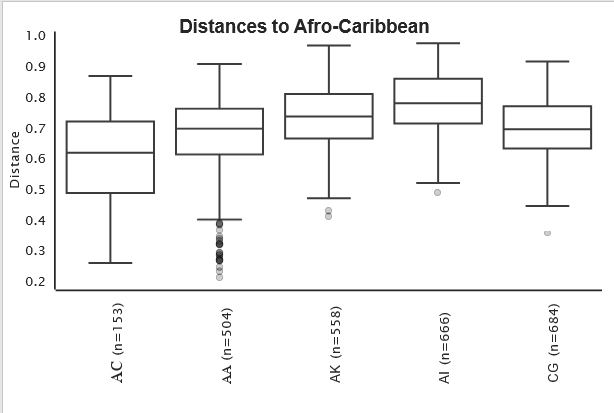


PERMANOVA (Pairwise), Test statistic: pseudo-F, N=152, No. of groups = 5, test statistic = 8.98093,

p-value = 0.001 No. of permutations = 999

| Group 1 | Group 2 | Sample size | Permutations | pseudo-F | p-value | q-value |
| --- | --- | --- | --- | --- | --- | --- |
| African American | African Kenyan | 59 | 999 | 13.0151614 | 0.001 | 0.00125 |
| African American | Afro-Caribbean | 46 | 999 | 13.05685969 | 0.001 | 0.00125 |
| African American | Asian Indonesian | 65 | 999 | 18.74450549 | 0.001 | 0.00125 |
| African American | Caucasian German | 66 | 999 | 16.56179386 | 0.001 | 0.00125 |
| African Kenyan | Afro-Caribbean | 49 | 999 | 5.710809759 | 0.001 | 0.00125 |
| African Kenyan | Asian Indonesian | 68 | 999 | 3.582042539 | 0.002 | 0.002222 |
| African Kenyan | Caucasian German | 69 | 999 | 2.766576067 | 0.005 | 0.005 |
| Afro-Caribbean | Asian Indonesian | 55 | 999 | 7.314320436 | 0.001 | 0.00125 |
| Afro-Caribbean | Caucasian German | 56 | 999 | 7.558312519 | 0.001 | 0.00125 |
| Asian Indonesian | Caucasian German | 75 | 999 | 8.112791419 | 0.001 | 0.00125 |
